# Specificity of surface EMG recordings for gastrocnemius during upright standing

**DOI:** 10.1038/s41598-017-13369-1

**Published:** 2017-10-16

**Authors:** Taian Martins Vieira, Alberto Botter, Silvia Muceli, Dario Farina

**Affiliations:** 1 Laboratorio di Ingegneria del Sistema Neuromuscolare (LISiN), Dipartimento di Elettronica e Telecomunicazioni, Politecnico di Torino, Torino, Italy; 2 Clinic for Trauma Surgery, Orthopaedic Surgery and Plastic Surgery, Research Department of Neurorehabilitation Systems, University Medical Center Göttingen, Göttingen, 37075 Germany; 3 Department of Bioengineering, Imperial College London, SW7 2AZ London, UK

## Abstract

The relatively large pick-up volume of surface electrodes has for long motivated the concern that muscles other than that of interest may contribute to surface electromyograms (EMGs). Recent findings suggest however the pick-up volume of surface electrodes may be smaller than previously appreciated, possibly leading to the detection of surface EMGs insensitive to muscle activity. Here we combined surface and intramuscular recordings to investigate how comparably action potentials from gastrocnemius and soleus are represented in surface EMGs detected with different inter-electrode distances. We computed the firing instants of motor units identified from intramuscular EMGs detected from gastrocnemius and soleus while five participants stood upright. We used these instants to trigger and average surface EMGs detected from multiple skin regions along gastrocnemius. Results from 66 motor units (whereof 31 from gastrocnemius) revealed the surface-recorded amplitude of soleus action potentials was 6% of that of gastrocnemius and did not decrease for inter-electrode distances smaller than 4 cm. Gastrocnemius action potentials were more likely detected for greater inter-electrode distances and their amplitude increased steeply up to 5 cm inter-electrode distance. These results suggest that reducing inter-electrode distance excessively may result in the detection of surface EMGs insensitive to gastrocnemius activity without substantial attenuation of soleus crosstalk.

## Introduction

Electromyograms (EMGs) may be recorded from within the muscle or from the skin surface. In the former case, EMGs are often used to study physiological properties of motor units (e.g., fatigability, recruitment threshold^1^). Classic bipolar surface EMGs, on the other hand, are expected to provide a global indication on the degree and timing of muscle activity^2,3^. Distinct applications for intramuscular and surface EMGs result from obvious differences in detection selectivity; in relation to surface EMGs, intramuscular recordings sample from a smaller fraction of the muscle volume. The low selectivity of surface electrodes has motivated the concern that surface EMGs are not specific for the muscle over which electrodes are positioned; i.e. they may sample from neighbour muscles as well. This volume conducted signal, referred to as crosstalk, may originate from deeper muscles (ankle flexors and extensors^4^), from muscles located in the nearby, transverse neighbourhood of the target muscle (vastus medialis and lateralis^5^) or from both (forearm muscles^6^). In the presence of crosstalk, estimates of the timing and the relative degree of activation of the target muscle may be inaccurate.

An issue opposite to crosstalk is the possibility that surface EMGs are not sensitive to muscle activation. The view that muscle activity may not be represented in EMGs is well accepted for intramuscular recordings^7,8^. Hodges and Gandevia^9^ have indeed advised to record from multiple locations when inferences on the behaviour of motor units are to be drawn from a single, selective pair of intramuscular electrodes. Perhaps because of the interferential pattern often observed in surface EMGs, similar considerations seem not applicable to surface recordings. Previous, classic accounts have however questioned whether EMGs detected from a single skin location would represent the activity of the whole volume of muscles such as the gastrocnemius^10,11^. And the recent use of arrays of electrodes has stimulated the growth of this seminal thought. Moreover, we have noticed that action potentials of single motor units have localised amplitude in bipolar surface EMGs detected along the gastrocnemius proximo-distal axis^12,13^. This observation leads to the question of whether sampling from a single, local skin site may provide a representative view of the entire muscle. As for crosstalk, inferences on the degree and timing of muscle activity may be inaccurate if EMGs are not sensitive to muscle activation.

**Figure 1:**
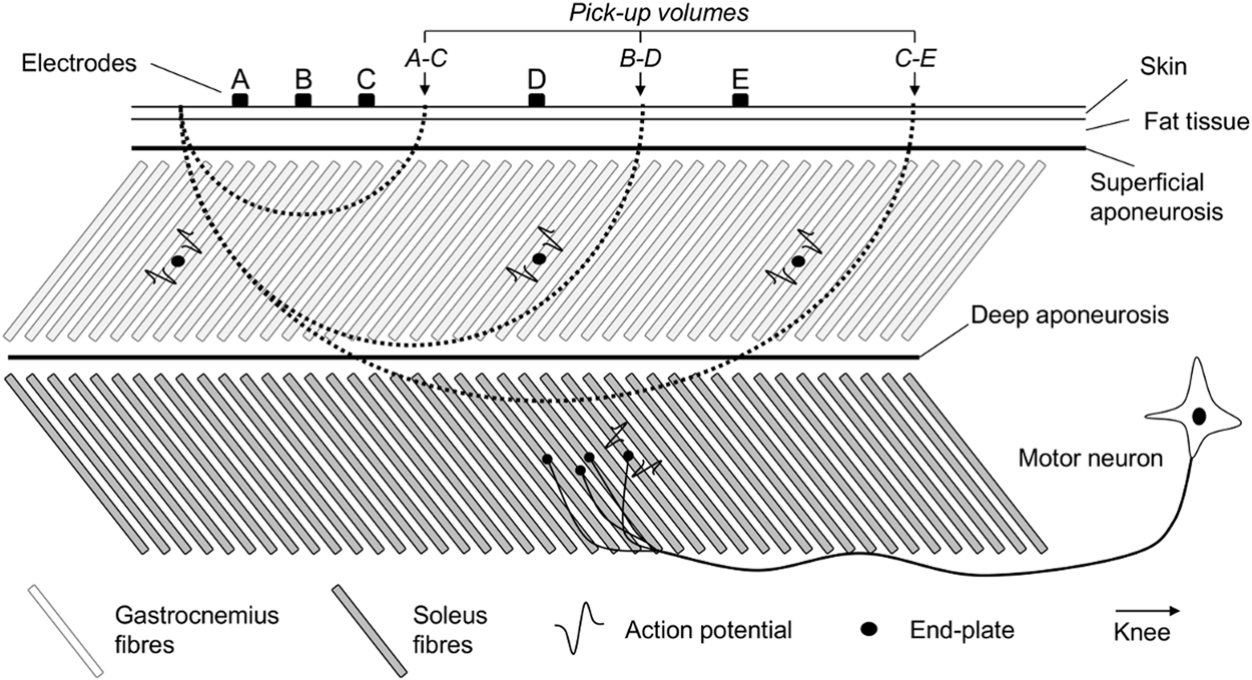
Pick-up volume and inter-electrode distance. A schematic representation of the tissue volume sampled by pairs of electrodes with different inter-electrode distances is shown. Dashed lines indicate the pick-up volume of individual pairs of electrodes; i.e., the region within which action potentials contribute substantially to the surface EMGs. By substantially we mean the amplitude of action potentials within the pick-up volume is at least 10% of that of action potentials located immediately below the recording electrodes^14,31^. Note the number of gastrocnemius fibres included in the pick-up volume increases with the inter-electrode distance. Surface EMGs detected by electrodes A and C sample predominantly from the most distal gastrocnemius fibres; unrepresentative muscle sampling. By increasing the inter-electrode distance (electrodes B-D), surface EMGs sample from more proximal gastrocnemius fibres. After a certain distance (cf. electrodes C-E in Fig. 1), fibres from the deep, soleus muscle are expected to contribute substantially to the surface EMG; EMGs are not specific for gastrocnemius.

In this study we combined intramuscular and surface recordings to investigate how similarly action potentials of both gastrocnemius and soleus motor units are represented in surface EMGs collected from gastrocnemius. We compared the amplitude of action potentials of motor units from these two muscles to determine the presence of action potentials generated by soleus motor units in recordings obtained with surface electrodes placed over gastrocnemius (soleus crosstalk) and the spatial localisation of action potentials of gastrocnemius motor units in surface recordings. Given the tissue volume sampled by the bipolar EMGs increases with the distance between electrodes^8,14,15^, we addressed these issues for a large range of inter-electrode distances. We hypothesised that by increasing inter-electrode distance the bipolar EMGs would better represent the activity of gastrocnemius motor units, at the cost of potentially decreasing the specificity of surface EMGs for gastrocnemius (i.e., increasing soleus crosstalk; Fig. 1). Henceforth we use the term representative to indicate the ability of representing all active, gastrocnemius motor units and the term specificity to indicate the surface EMG collected from gastrocnemius contains (if) negligible contribution from soleus. The ratio between the amplitude of action potentials of soleus (crosstalk) and gastrocnemius (representativeness) motor units as a function of inter-electrode distance quantifies the relative importance of representation and crosstalk in surface EMG.

## Methods

### Participants and experimental procedures

Five healthy men (age range: 26–37 years; stature: 170–189 cm; body mass: 70–80 kg) participated to this study after providing written informed consent. The experimental protocol was approved by the ethic committee of the University Medical Center Goettingen, Germany. All experimental procedures were performed according to the Declaration of Helsinki.

Calf muscles’ activity was detected while participants stood at ease. Subjects were asked to stand naturally for 60 s on a force platform, with their arms hanging loosely alongside the body and their feet in a comfortable position. No gross movements of legs, arms and trunk were allowed during experiments. Three trials were applied per subject, with 5 min rest periods in-between. The trial providing EMGs of greatest quality, with the highest number of clearly visible action potentials in the intramuscular recordings, was retained for analysis. It was our interest in the active control of bipedal standing that motivated our decision to focus the analysis on the motor units active during standing, hereafter referred to as postural motor units. Moreover, the identification of individual motor units in both intramuscular and surface EMGs is facilitated during standing^13^, since this condition demands slight, active loading of the calf muscles^10,16^.

### Electromyographic recordings

**Figure 2:**
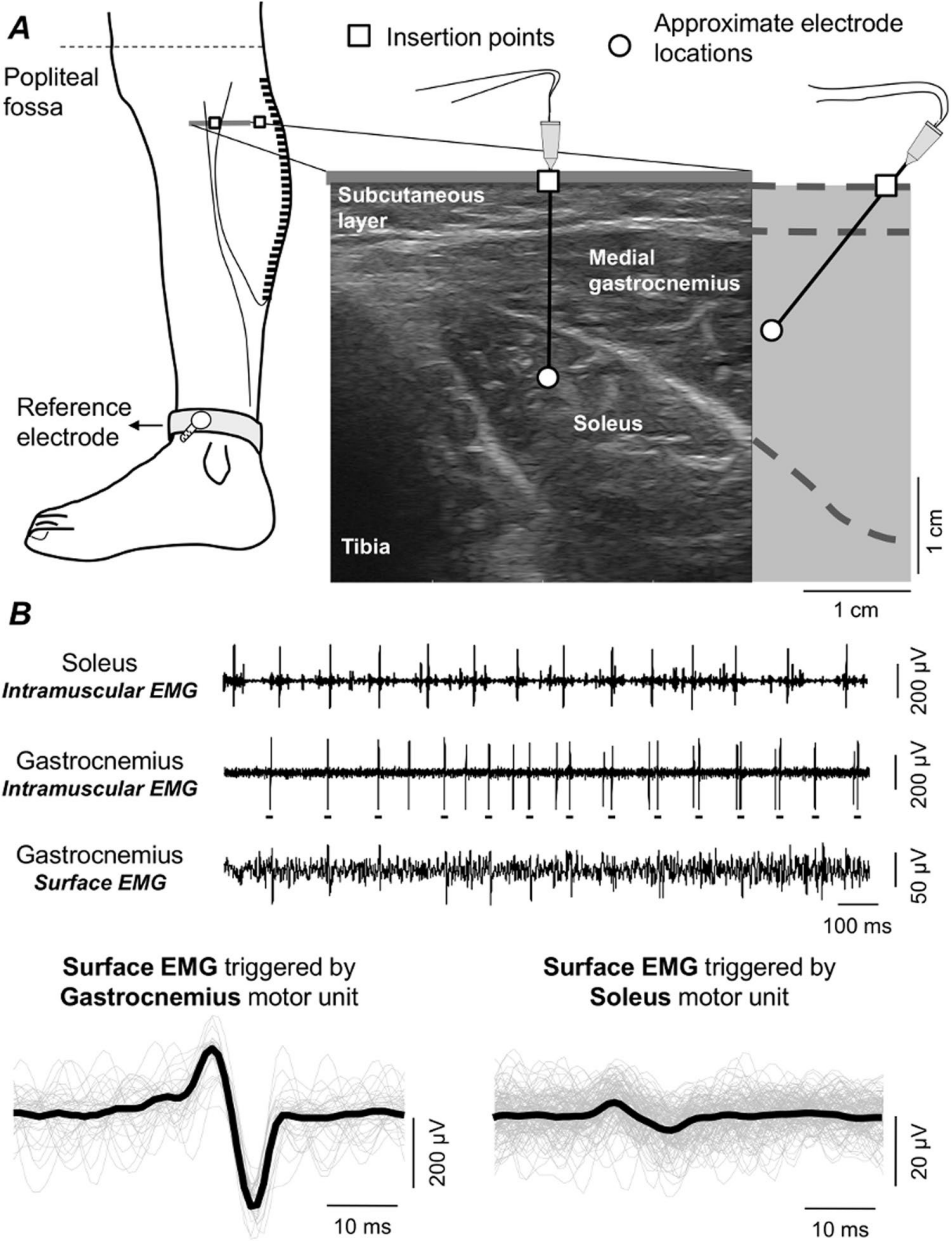
Surface and intramuscular EMG detection and analysis. (**A**) schematic illustration of the positioning of intramuscular and surface electrodes. Almost the whole gastrocnemius muscle was covered proximo-distally by 32 surface electrodes. With the guidance of ultrasound scanning (right panel), wire electrodes were inserted into gastrocnemius and soleus, roughly beneath the parasagittal section defined by the surface electrodes. (**B**) examples of intramuscular EMGs collected from both muscles and a single, differential surface recording (5 mm inter-electrode distance) are shown. Grey traces in the bottom left correspond to short (60 ms) epochs of surface EMGs triggered with the firing instants of a motor unit identified from the gastrocnemius, intramuscular EMG (cf. short horizontal bars underneath large spikes). These epochs were then averaged, producing the spike, triggered average representation of the motor unit action potential in the surface EMG (black trace; cf. Methods). An example of the surface representation of a motor unit from soleus is shown in the right bottom panel.

Intramuscular and surface EMGs were detected concurrently, amplified and then sampled at 10 kHz with a 12 bit A/D converter (EMG-USB2, OT-Bioelettronica, Italy). Intramuscular EMGs were obtained from the soleus and medial gastrocnemius muscles of the right leg with stainless steel, fine wire electrodes (0.2 mm diameter). While subjects were laying over their right side, two wires were inserted into each muscle with a 25-gauge, 25 mm long hypodermic needle. Monopolar surface EMGs were detected along the medial head of gastrocnemius using four linear arrays of 8 electrodes, each with 5 mm inter-electrode distance. After shaving the recording site and cleaning the skin with abrasive paste, arrays were placed serially along the muscle. The distance between the most distal and proximal electrodes of consecutive arrays was kept at 5 mm, resulting in a linear array of 32 electrodes with 5 mm inter-electrode distance (Fig. 2A). The most proximal surface electrode was located as close as possible to the popliteal fossa and the ground electrode was placed at the medial malleolus. The calf was scanned with an ultrasound probe (Echo Blaster, Telemed UAB, Lituania) to determine subcutaneous and muscle thicknesses and to guide electrodes’ positioning. Wires were inserted obliquely into the two calf muscles to ensure the tip of the wires was located roughly below the surface electrodes (Fig. 2A). Insertion depth varied for both muscles, depending on the thickness of gastrocnemius and subcutaneous tissues. Soleus wire electrodes were inserted medially, where gastrocnemius thickness was smallest (Fig. 2A).

### Quantifying the surface representation of single motor units

Firings of individual motor units were identified from both intramuscular and surface recordings. First, the bipolar, intramuscular EMGs were decomposed into the constituent trains of motor unit action potentials using the software EMGLAB^17^. Instants of firing of individual motor units were used to trigger and then average the surface EMGs. This procedure, known as spike-triggered averaging^18^, provides the surface representation of action potentials of single motor units, as schematically illustrated in Fig. 2B for a single bipolar, surface EMG. The surface representation of action potentials was assessed for motor units identified from both the soleus and the gastrocnemius muscles. Given the pick-up volume of intramuscular electrodes is relatively small, we further decomposed the surface EMGs using an automated, validated procedure^19^ to obtain data from as many gastrocnemius motor units as possible.

The surface representation of motor units from soleus and gastrocnemius muscles was assessed for different inter-electrode distances. Bipolar, surface EMGs (*bEMG*) were computed as the algebraic difference between all possible combinations of monopolar signals:

1 $$\begin{array}{c}bEM{G}_{i,k}=staEM{G}_{i}-staEM{G}_{i+k}\\ k=1,...,d-1\\ i=1,...,d-k\\ k=\frac{{\rm{i}}{\rm{n}}{\rm{t}}{\rm{e}}{\rm{r}}-{\rm{e}}{\rm{l}}{\rm{e}}{\rm{c}}{\rm{t}}{\rm{r}}{\rm{o}}{\rm{d}}{\rm{e}}{\rm{d}}{\rm{i}}{\rm{s}}{\rm{t}}{\rm{a}}{\rm{n}}{\rm{c}}{\rm{e}}}{0.5\,{\rm{c}}{\rm{m}}}\end{array}$$

where *staEMG*_*i*_ corresponds to the spike triggered averaged monopolar signal detected by the surface electrode *i*, starting from the most proximal electrode. The inter-electrode distance varied at 5 mm steps, from the smallest inter-electrode distance (5 mm) to the distance between the most proximal electrode and the electrode (*d*) located at or just proximally to the distal extremity of the gastrocnemius superficial aponeurosis. Electrodes located distally to the superficial aponeurosis were excluded because gastrocnemius thickness decreases progressively from the distal extremity of its superficial aponeurosis to its junction with the Achilles tendon. Consequently, the shortest distance between electrodes located in this region and the soleus muscle decreases as well. Moreover, surface EMGs detected over the superficial aponeurosis and over the very distal gastrocnemius region provide different information, as in the former and latter case electrodes are aligned respectively obliquely and parallel to gastrocnemius fibres (cf. Fig. 1 in Hodson-Tole *et al*.^20^).

### Assessing soleus crosstalk and the representativeness of gastrocnemius motor units in the surface EMGs

**Figure 3:**
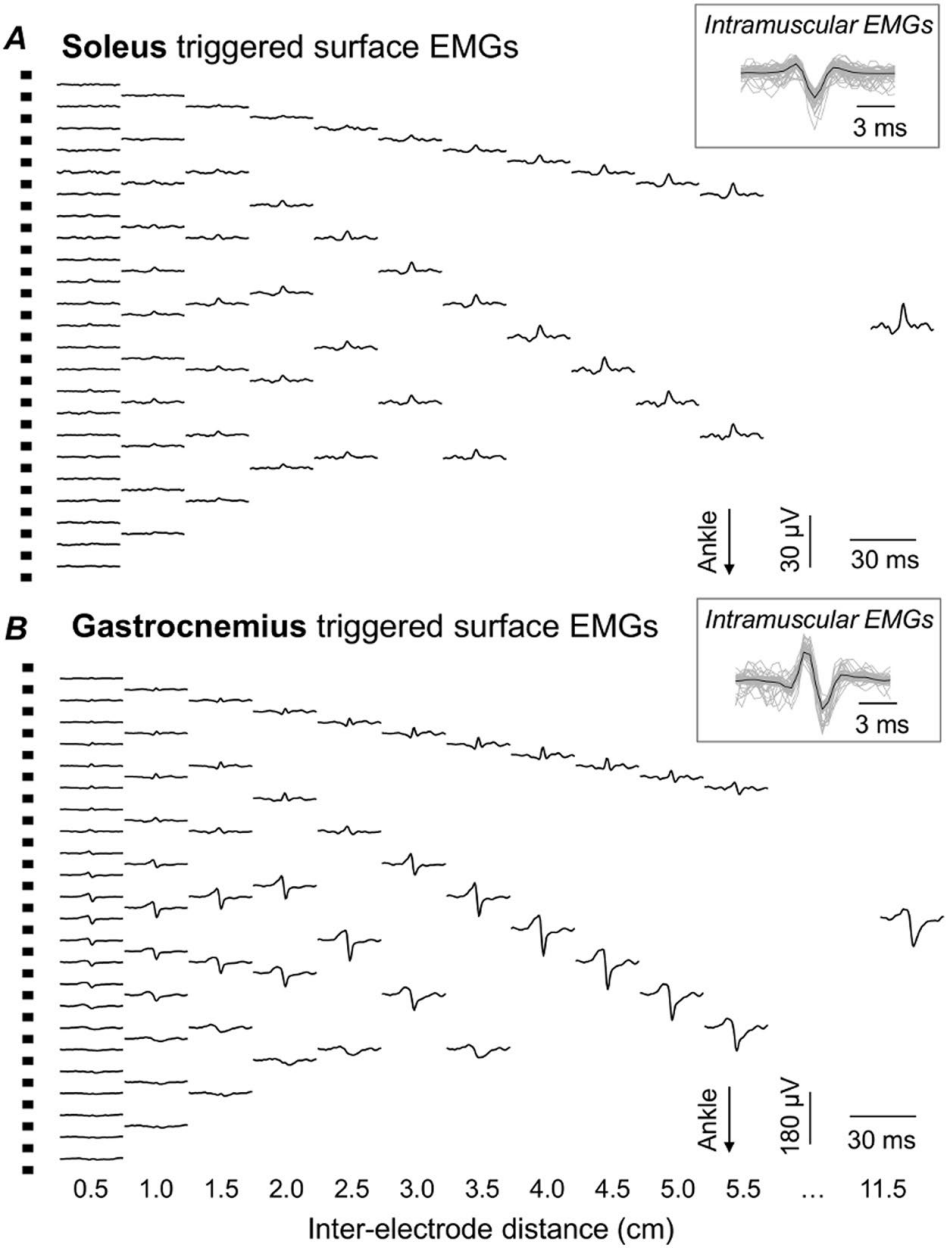
Inter-electrode distance and the surface representation of motor units. Spike triggered and averaged EMGs (Fig. 2B) are shown for two motor units identified for a single participant. Traces in panel A correspond to EMGs triggered and averaged by the firing instants of a motor unit identified from the soleus, intramuscular EMG (cf. inset), for different inter-electrode distances. The same information is shown in panel B for a motor unit identified from the gastrocnemius, intramuscular EMG. For clarity, EMGs for only 12 out of the 23 inter-electrode distances considered for this participant are shown. Note the different scales used to represent soleus and gastrocnemius, surface EMGs.

The root mean square (RMS) amplitude was considered to assess soleus crosstalk in the surface EMGs. RMS values were computed for all combinations of bipolar EMGs (equation (1)), separately for each inter-electrode distance, pair of electrodes and muscle. This procedure provided different numbers of RMS values per inter-electrode distance. For example, for the participant whose data is shown in Fig. 3, 23 RMS values were obtained for the 0.5-cm inter-electrode distance whereas the largest inter-electrode distance overtly provided a single RMS value. The 90^th^ percentile of the distribution of RMS values obtained for each inter-electrode distance was considered to provide a single value representing the contribution of individual motor units to the surface EMG; for the largest inter-electrode distances, each providing less than five RMS values, the median RMS value was used. It should be noted this procedure is conservative, as the resulting RMS values do not depend on whether greatest surface potentials are detected locally or sparsely on the skin. Since soleus surface potentials were of very low amplitude for short inter-electrode distances (see Results), averaging RMS values for any groups of locally distributed electrodes could possibly underestimate their contribution to the surface EMG. Finally, the RMS amplitude obtained for each soleus motor unit was normalised with respect to the RMS amplitude averaged across all gastrocnemius motor units, separately for each inter-electrode distance. This normalised RMS value provides a relative estimate of soleus crosstalk on surface EMGs detected from gastrocnemius. High crosstalk indicates that surface EMG detected over gastrocnemius is not specific for gastrocnemius.

**Figure 4:**
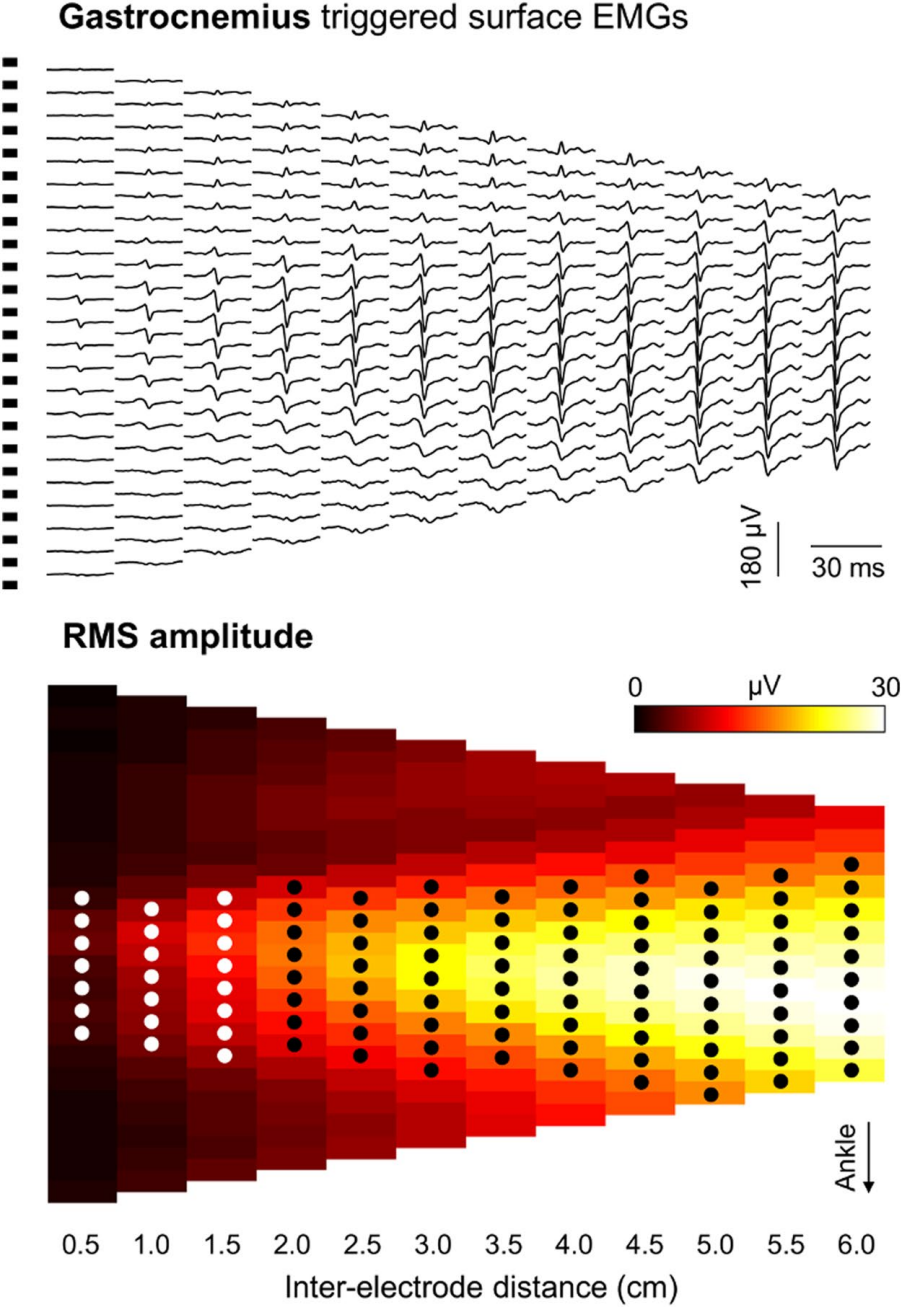
Assessing the surface representation of gastrocnemius motor units. Raw surface potentials and their Root Mean Square (RMS) amplitude are shown in panels A and B respectively. Surface potentials were obtained by triggering and averaging surface EMGs with the firing instants of a motor unit obtained by decomposing the intramuscular EMG detected from the gastrocnemius muscle of a representative participant. Surface potentials and RMS values are shown for bipolar electrodes centred at all possible skin locations, separately for inter-electrode distances ranging from 0.5 to 6.0 cm. Circles indicate the bipolar electrodes providing surface potentials with RMS amplitude greater than 50% of the maximal amplitude obtained for each of the 12 inter-electrode distances considered.

Similarly, RMS values were considered to assess the representativeness of gastrocnemius motor units in the surface EMGs (Fig. 4). We specifically computed the number of channels providing gastrocnemius surface potentials with RMS amplitude greater than 50% of the maximal amplitude, separately for each of the first 12 inter-electrode distances (from 0.5 to 6.0 cm; circles in Fig. 4). We selected the first 12 distances because it corresponded to about half of the greatest inter-electrode distance obtained for all participants and because we show below the amplitude of action potential of gastrocnemius units does not increase for inter-electrode distances greater than ~4.5 cm. We then normalised the number of channels with high RMS amplitude with respect to the total number of channels, providing an indication on the relative size of the skin region where greatest surface potentials were detected from gastrocnemius and for each inter-electrode distance. If gastrocnemius motor units are represented equally everywhere on the skin, all channels should detect surface potentials with similar RMS amplitude, regardless of the inter-electrode distance considered. Conversely, according to Fig. 1, the relative number of channels detecting relatively large potentials should increase with the inter-electrode distance if individual gastrocnemius units are represented locally on the skin. It should be noted the most proximal and distal electrodes respectively for the most proximally and distally centred pair of electrodes covered the same gastrocnemius region, for all 12 inter-electrode distances considered.

### Statistics

The confidence interval of the distribution of normalised RMS values obtained for short inter-electrode distance was calculated to assess the effect of inter-electrode distance on soleus crosstalk. As illustrated below for all participants, the normalised RMS values decreased consistently for *shortest inter-electrode distances*, from 0.5 cm to about 2.0 cm. These smallest RMS values were therefore regarded as a reference for the minimal possible soleus crosstalk (see Discussion). Confidence intervals were computed from all normalised RMS values provided by the *shortest inter-electrode distances*, separately for each subject. Significant increases in soleus crosstalk was verified for inter-electrode distances providing normalised RMS values greater than the upper bound of confidence intervals (i.e., *crosstalk thresholds*). Spearman correlation was considered to assess any dependence of these inter-electrode distances on the thickness of gastrocnemius and subcutaneous tissues. Finally, Spearman correlation was applied to test for whether the relative number of channels providing greatest gastrocnemius surface potentials is associated with the inter-electrode distance.

## Results

### Motor units

A total of 66 motor units were identified from both gastrocnemius and soleus muscles. Thirty five units were decomposed from soleus intramuscular EMGs whereas 31 units were obtained through decomposition of gastrocnemius intramuscular and surface EMGs. The median number of motor units identified per subject was 6 (range: 5–8) for gastrocnemius and 7 (5–9) for soleus. Visual inspection revealed none of the motor units identified were represented concurrently in the spike triggered average EMGs detected intramuscularly from both muscles.

### The surface representation of soleus and gastrocnemius motor units

Surface potentials with different amplitudes were observed for different muscles and inter-electrode distances. A representative example is illustrated in Fig. 3 for one soleus and one gastrocnemius motor unit. Surface EMGs detected with *shortest inter-electrode distances* were almost flat when triggered and averaged with the firing instants of the soleus motor unit (Fig. 3A). The amplitude of the surface representation of these soleus potentials became progressively higher as inter-electrode distance increased, being the highest for the largest inter-electrode distance (cf. 11.5 cm EMGs in Fig. 3A). Even though bigger soleus potentials were observed for greater inter-electrode distances, their amplitude was smaller than 30 uV_pp_. On the other hand, action potentials from the gastrocnemius motor unit were already distinguishable from the background noise even for the *shortest inter-electrode distances*, at the central muscle region (cf. EMGs for 0.5 cm inter-electrode distance in Fig. 3B). Differently from soleus, the gastrocnemius surface potentials did not increase progressively for the entire range of inter-electrode distance; their amplitude did not increase for distances greater than ~4 cm.

**Figure 5:**
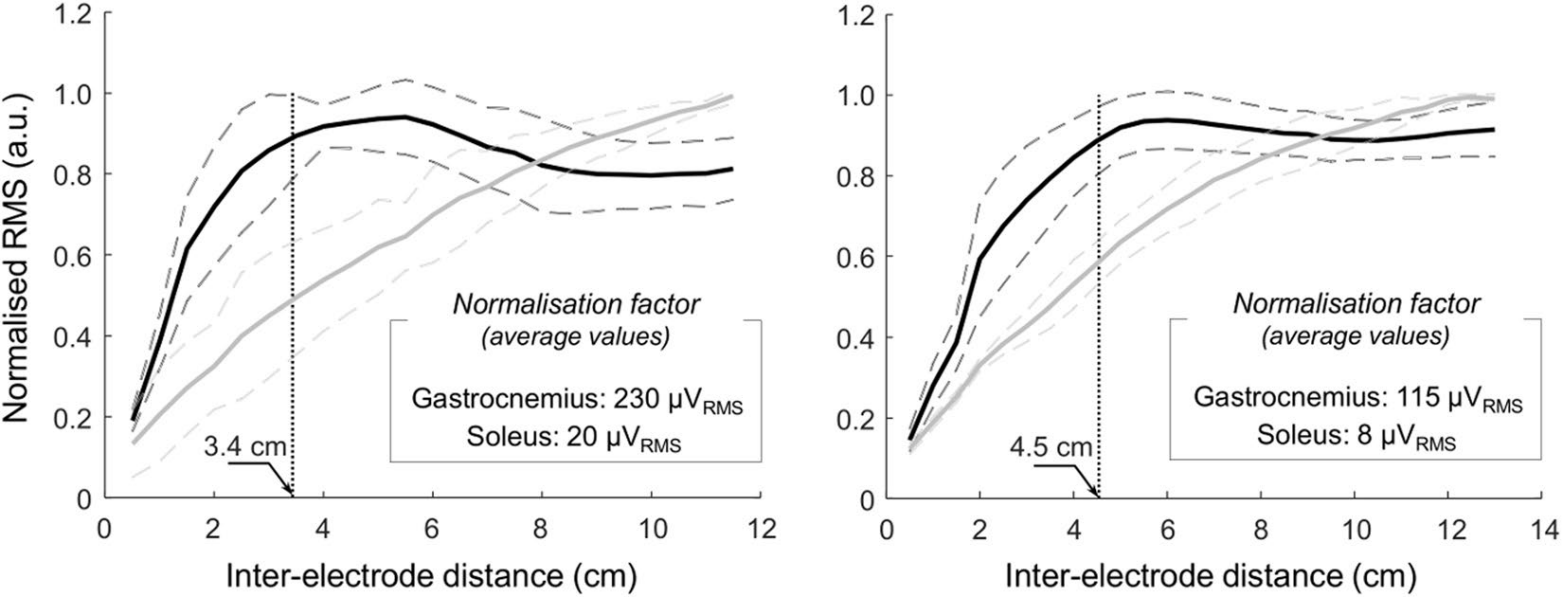
Root mean square amplitude of soleus and gastrocnemius surface EMGs. Mean (continuous lines) and standard deviation (dashed lines) are shown for the root mean square (RMS) amplitude of surface EMGs triggered by the firing instants of motor units identified from the gastrocnemius (black lines) and soleus (grey lines) muscles. Amplitude values are shown for all inter-electrode distances considered for two participants, ranging from 0.5 cm to 11.5 cm (left panel) and from 0.5 cm to 13.0 cm (right panel). For clarity, RMS values were normalised with respect to the absolute maximal RMS value across motor units and inter-electrode distances, separately for gastrocnemius and soleus muscles. The vertical dotted line indicates the inter-electrode distance for which the mean RMS amplitude of gastrocnemius triggered EMGs reaches 90% of its maximum.

Qualitative differences between soleus and gastrocnemius surface potentials with inter-electrode distance could be well appreciated by comparing their RMS amplitude. Figure 5 shows the RMS amplitude of surface potentials averaged across all motor units identified from soleus and gastrocnemius of two participants. While the RMS amplitude of soleus surface potentials increased linearly with inter-electrode distance for both subjects, the amplitude of gastrocnemius potentials did not. RMS values obtained for gastrocnemius units increased steeply for small increases in inter-electrode distance, roughly reaching a plateau region after a certain, variable inter-electrode distance between subjects (cf. dotted, vertical lines in Fig. 5). The distance after which gastrocnemius surface potentials reached 90% of the maximum amplitude ranged from 2.9 to 4.5 cm across the five subjects tested.

### Inter-electrode distance and soleus crosstalk

**Figure 6:**
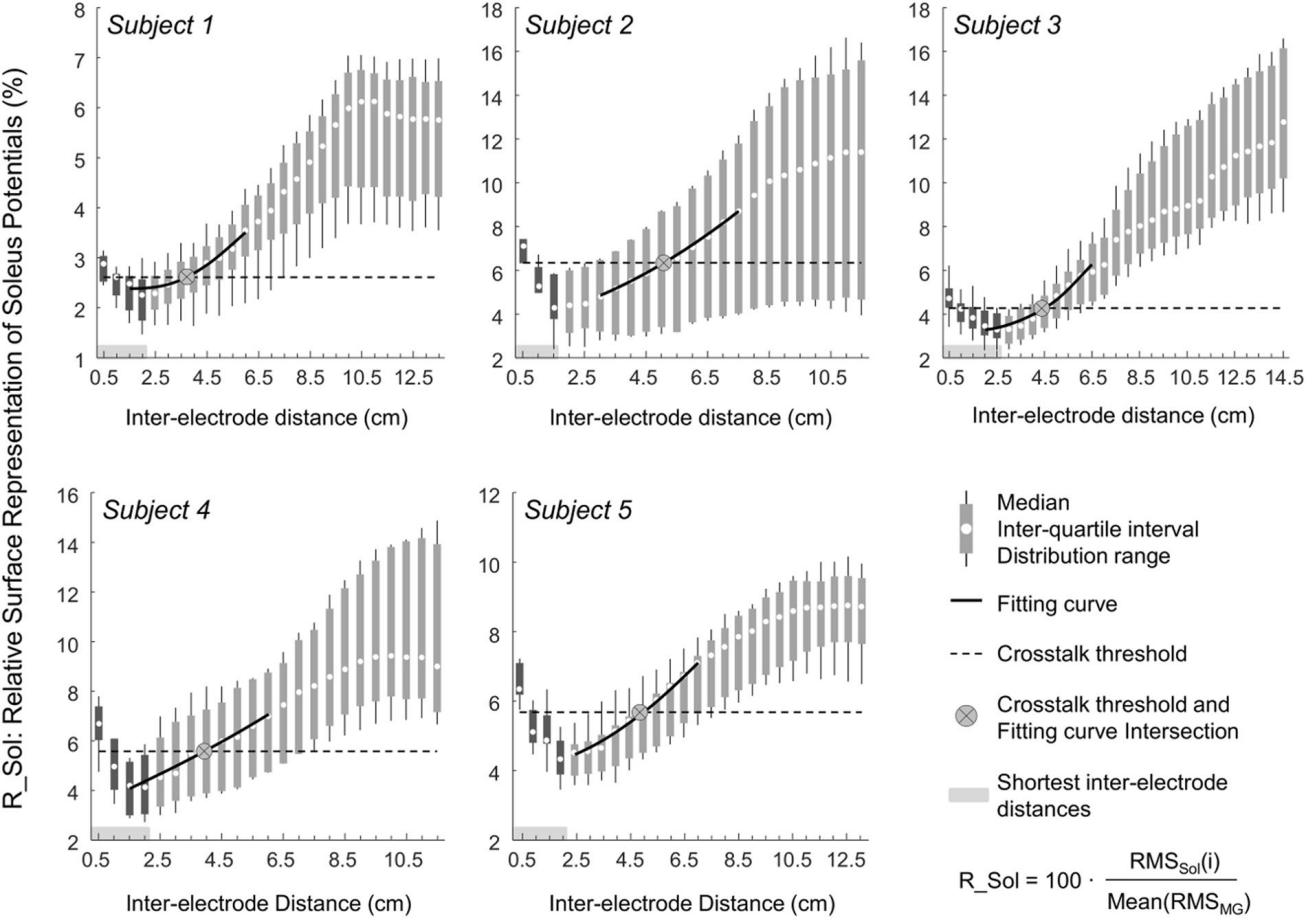
Relative, surface representation of soleus motor units. The distribution of the amplitude of surface EMGs, triggered and averaged with the firing instants of soleus motor units, is shown for each participant and for each inter-electrode distance. These amplitude values were normalised by the amplitude of surface EMGs triggered with the firing instants of gastrocnemius motor units, averaged across all firings of all gastrocnemius motor units identified for each subject and separately for each inter-electrode distance. The horizontal, shaded rectangles indicate the *shortest inter-electrode distances* considered to compute the *crosstalk threshold* (dashed, horizontal lines; cf. *Statistics*). The crossed circle corresponds to the intersection between the *crosstalk threshold* and the third order polynomial fitting the 10 median values nearest to the *crosstalk threshold*. Different scales have been used to take into account the different distribution range across subject, which was likely due to the small sample of units detected during the standing task.

**Figure 7:**
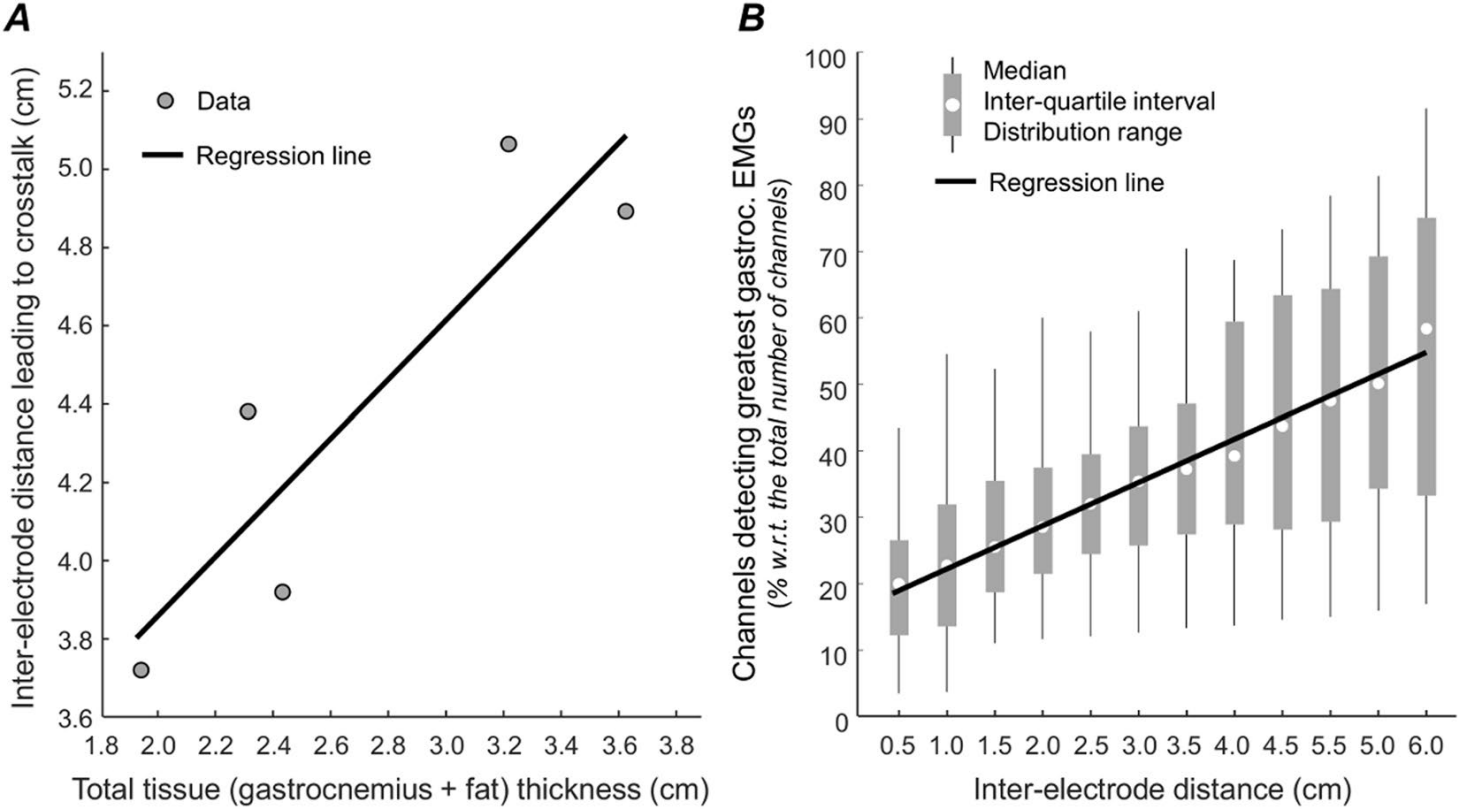
Tissue thickness and the surface representation of gastrocnemius units. A scatter plot with data obtained from the five subjects tested is shown in (**A**). The total tissue thickness (subcutaneous plus gastrocnemius thickness) is plotted in the abscissa. The inter-electrode distance over which soleus motor units provided a significant contribution to the surface EMGs (Fig. 5) is shown in the ordinate. Spearman *Rho* and its significance level were respectively 0.89 and 0.11. (**B**) shows the distribution of the relative number of channels detecting gastrocnemius surface potentials with amplitude greater than 50% of the maximal amplitude across all subjects and motor units, separately for each inter-electrode distance from 0.5 to 6.0 cm. Spearman analysis indicates a significant correlation between the median, relative number of channels and inter-electrode distance (*Rho* = 0.98; *P* < 0.001). Continuous black lines (regression lines) were drawn to indicate the positive trend observed for the data shown in both panels.

When considering group data, the relative contribution of soleus motor units to the surface EMGs depended on the inter-electrode distance. In relation to the RMS amplitude of surface EMGs triggered by gastrocnemius units for the *shortest inter-electrode distances*, the RMS amplitude of surface EMGs triggered by soleus motor units amounted to 4.2 ± 1.6% (mean ± st. dev.; Fig. 6; *N* = 141; number of soleus motor units multiplied by number of *shortest inter-electrode distances* and summed over subjects). The *crosstalk threshold*, over which the amplitude of soleus surface potentials increased at a significantly greater rate than that of gastrocnemius potentials, ranged therefore from 2.6% to 6.4% across subjects (cf. horizontal, dashed lines in Fig. 6). Consequently, the inter-electrode distance above which the degree of soleus crosstalk started to increase varied from 3.7 to 5.0 cm between individuals, as indicated by the x coordinate of crossed circles in Fig. 6. This critical, inter-electrode distance was not significantly correlated with the total subcutaneous and gastrocnemius thickness (Spearman Rho = 0.89; *P* = 0.11; *N* = 5 subjects), notwithstanding the clear positive trend observed between both variables (Fig. 7A).

### Inter-electrode distance and the representativeness of gastrocnemius motor units

The median, relative number of channels providing greatest gastrocnemius surface potentials increased with the inter-electrode distance (Fig. 7B; Spearman *Rho* = 0.98; *P* < 0.001; *N* = 12 inter-electrode distances). Across all motor units identified from intramuscular and surface EMGs detected from gastrocnemius, surface potentials with amplitude greater than 50% of the maximal amplitude were detected by ~20% of the 23 locations where bipolar electrodes spaced by 0.5 cm could be positioned. This figure roughly trebled when considering bipolar electrodes with 6.0 cm inter-electrode distance (Fig. 7B).

## Discussion

Intramuscular and surface EMGs were sampled to investigate how comparably action potentials of soleus and gastrocnemius motor units may be represented in surface recordings from the gastrocnemius muscle during standing. Different centre-to-centre distances between consecutive, surface electrodes were assessed. Results from five participants revealed that, in relation to gastrocnemius, the amplitude of action potentials of soleus motor units: i) amounted to less than 6.4% for surface EMGs sampled with inter-electrode distances smaller than 5 cm; ii) increased progressively with inter-electrode distance, reaching ~10% for large inter-electrode distances (8.5 cm). Our results further show that smaller inter-electrode distances were less likely to sample action potentials from individual, gastrocnemius motor units. These results suggest the surface EMGs sample from a relatively small fraction of the gastrocnemius muscle, especially if detected by individual pairs of closely spaced electrodes.

Different experimental protocols have been applied to assess EMG crosstalk. The most simplistic approach consists in evaluating EMGs collected during selective voluntary muscle contractions^6^. For example, EMGs detected from tibialis anterior during voluntary, plantar flexions are expected to reflect mainly the activity of the ankle plantar flexors. However, co-activation of the antagonist muscle likely occurs and the signal recorded at the tibialis anterior cannot be attributed to crosstalk only^21^. A more controlled means of studying crosstalk is based on electrical stimulation; monopolar, stimulation pulses delivered proximally to tibialis anterior were observed to result in the detection of surface potentials almost everywhere on the leg^4^. Nonetheless, ascertaining the exclusive recruitment of target muscles in voluntary and electrically elicited contraction is not possible. Certainty of exclusive recruitment demands the isolation of nerve branches serving the target muscle^7,8^, which is not feasible. Here we circumvented this issue. We triggered and averaged surface EMGs separately with the firing instants of soleus and gastrocnemius motor units^18^. Instances of synchronised firings between units are sporadic and thus do not contribute to the triggered, averaged representation of motor units of either muscle. Moreover, none of the 66 motor units identified was represented concurrently in intramuscular EMGs collected from both muscles. We feel therefore safe to state the surface representation of soleus motor units was assessed without interference from gastrocnemius motor units and *vice versa*.

Action potentials from soleus motor units were not generally well represented in surface recordings from gastrocnemius. For the shortest electrodes’ spacing (0.5 cm), the amplitude of soleus surface potentials amounted to ~5% of that of gastrocnemius potentials. Increasing the inter-electrode distance by small values resulted in soleus potentials progressively smaller than gastrocnemius potentials (Fig. 6), not because smaller soleus potentials were detected for inter-electrode distances slightly greater than 0.5 cm but because gastrocnemius surface potentials increased steeply with small increases in inter-electrode distance (Fig. 5). We therefore considered these *shortest inter-electrode distances* as a reference condition determining the statistical significance of soleus crosstalk in surface EMGs (Fig. 6; see Methods). This decision was further motivated by the well accepted notion that more closely spaced electrodes sample from smaller muscle volumes^14,15^ and, most importantly, by the fact that action potentials of all soleus motor units were less clearly represented for shorter inter-electrode distances (e.g. Fig. 3A). Our results show there is a certain inter-electrode distance, ranging from ~3.7 to ~5.0 cm, after which soleus crosstalk started to increase. Within this critical distance, soleus crosstalk did not exceed 6.4% and distances smaller than this critical value did not attenuate soleus crosstalk (Fig. 6). These crosstalk estimates are somewhat smaller than those reported by others in humans for similar inter-electrode distances^4,15,22^. The key methodological difference between current and previous studies is the assessment of crosstalk at the motor unit level. Previously, crosstalk was assessed during either voluntary or electrically elicited contractions. We, conversely, used intramuscular EMGs to isolate the contribution of motor units from both muscles to surface recordings, ensuring no mutual interference on the triggered, averaged EMGs (Figs 2–3). The crosstalk figures obtained here are indeed similar to those reported for animal studies, which assessed crosstalk by cutting the nerve serving target muscles^7,8^. In addition to showing crosstalk values smaller than those typically reported in the literature, our results indicate that reducing inter-electrode distances below a certain value may not attenuate soleus crosstalk.

The amplitude of soleus and gastrocnemius surface potentials increased to different extents with inter-electrode distance (Figs 3–5). This differential surface representation between muscles is presumably associated with the number of fibres within the electrodes’ pick-up volume. It is well established that the contribution of distant sources increases with the inter-electrode distance, regardless of whether EMGs are detected intramuscularly or not^14,23^. Not surprisingly, indeed, the amplitude of intramuscular and surface, bipolar EMGs increases with the distance between electrodes^8,22,24,25^. It seems therefore reasonable to consider a progressively greater number of muscle fibres was included in the detection volume of more largely spaced electrodes (Fig. 1). Once most fibres of individual motor units were included in the pick-up volume of surface electrodes, further increasing inter-electrode distance would not be expected to provide action potentials with greater amplitude. While the relatively small increase of soleus, surface potentials suggests a potentially small soleus fraction was sampled by largely spaced electrodes, the amplitude plateau observed for all 31 gastrocnemius motor units (e.g. Fig. 5) suggests most fibres of gastrocnemius units were sampled by ~4 cm spaced electrodes. This observation is consistent with our previous findings on the spatial distribution of the amplitude of surface potentials of individual gastrocnemius units^13^ and is not in contrast with the possibility of spatially localised muscle units having relatively large territories^26,27^. This observation further suggests that closely spaced electrodes provide selective EMGs, potentially unrepresentative of gastrocnemius activity. If gastrocnemius units were represented equally everywhere on the skin, bipolar electrodes would detect surface potentials with similar amplitude, regardless of their location on the muscle. As shown in Figs 5 and 7B, action potentials of gastrocnemius motor units were however represented in relatively small skin regions. When sampled by 0.5-cm spaced bipolar electrodes, for example, gastrocnemius action potentials were represented in a region spanning ~20% of the whole skin region where these shortly spaced bipolar electrodes could have been centred (Fig. 7B). By increasing the distance between electrodes we observed surface potentials with similarly high amplitude were detected at a relatively greater skin region, spanning roughly 60% of the skin region available for surface recording from gastrocnemius (Fig. 7B). These results suggest greater inter-electrode distance leads to the detection of more representative surface EMGs.

Inferences on the whole muscle level may not proceed from unrepresentative EMGs. Ascertaining a given muscle is inactive or irresponsive to a given stimulus may not be possible, for example, from EMGs detected by closely spaced electrodes (e.g., Fig. 3B). This is well illustrated by Hodges and Gandevia^9^ for the diaphragm muscle. These authors observed a decrease in the amplitude of intramuscular EMGs during inspiration, likely because the active diaphragm fibres moved away from the electrodes. Similarly, EMGs recorded by more selective leads seem to be more sensitive to variations in electrodes’ position^2,28^. Since current and previous results^12,13^ show that surface EMGs sampled locally may not represent the whole gastrocnemius muscle (Figs 3B and 7B), the key question here is: how can we ensure the detection of surface EMGs representative of gastrocnemius activity and specific at the same time, i.e. with minimal crosstalk from soleus? Appropriately spacing surface electrodes may address this issue. Here we show that: i) the amount of soleus crosstalk did not decrease for inter-electrode distances smaller than a few centimetres; ii) gastrocnemius potentials increased steeply with small increases in inter-electrode distance; iii) more largely spaced surface electrodes are more likely to detect action potentials of individual gastrocnemius units. Excessively reducing inter-electrode distance may therefore lead to the detection of remarkably small gastrocnemius potentials while attenuating soleus crosstalk to negligible extents. In virtue of the variable subcutaneous and muscle thickness across subjects, there was not a single range of inter-electrode distances for which soleus crosstalk did not change (Fig. 7A). Nevertheless, cautiously increasing inter-electrode distance may lead to a more representative and thus reliable recording of gastrocnemius activity. According to results presented in this study, inter-electrode distances ranging from 3.5 to 5.0 cm seem to provide surface EMGs sensitive to action potentials generated within 40–50% of the gastrocnemius proximo-distal region (Fig. 7B) with negligible crosstalk from soleus (Fig. 6).

In this study, the surface representation of action potentials of gastrocnemius and soleus motor units was assessed during quiet standing. Small, postural motor units are expected to be recruited in this condition^10,29^. It is therefore relevant to question whether results reported here may extend to conditions imposing greater, active muscle loading. One may argue the ratio values reported in Fig. 6 would have been greater had we analysed motor units recruited in conditions more demanding than standing. The relevance of this problem is however likely reduced by our normalisation procedure. Crosstalk from soleus was quantified in relation to the amplitude of action potentials from gastrocnemius motor units. If larger units from both muscles were recruited during a given contraction, the relative amount of soleus crosstalk would possibly change by marginal amounts. Indeed, results from motor units of different sizes in the cat revealed the amount of soleus crosstalk increased by only ~2% when the plantar flexion force elicited increased from 10% to 100%^7^. Another potentially relevant issue to mention here is the electrode size. Given the size of electrodes has been shown to affect the amplitude of the recorded signal^30^, we understand it may affect both the representation of gastrocnemius action potentials in the surface EMG and the degree of soleus crosstalk. However, it should be noted our reasoning concerns the relationship between pick-up volume and inter-electrode distance, which holds irrespective of the electrode size (see Fig. 1). Even though we value the importance of assessing the surface representation of motor units of different sizes and the effect of electrode size, having evaluated exclusively the motor units recruited during standing for a fixed electrode size does not discredit our message. The choice of inter-electrode distance within a range of few centimetres influences substantially the spatial location of the recording and therefore the level of representativeness of the recording and less so the level of crosstalk.
